# A review of proposed mechanisms for neurodegenerative disease

**DOI:** 10.3389/fnagi.2024.1370580

**Published:** 2024-10-08

**Authors:** Benjamin M. Kelser, Eric M. Teichner, Robert C. Subtirelu, Kevin N. Hoss

**Affiliations:** ^1^School of Medicine, Case Western Reserve University, Cleveland, OH, United States; ^2^Sidney Kimmel Medical College (SKMC), Philadelphia, PA, United States; ^3^Department of Radiology, Perelman School of Medicine, University of Pennsylvania, Philadelphia, PA, United States

**Keywords:** neurodegenerative disease, synaptic spread hypothesis, selective vulnerability hypothesis, protein aggregation, tau, alpha-synuclein, TDP-43, neuroimaging advances

## Abstract

Neurodegenerative diseases, such as Alzheimer’s, Parkinson’s, and amyotrophic lateral sclerosis (ALS) affect millions and present significant challenges in healthcare and treatment costs. The debate in the field pivots around two hypotheses: synaptic spread and selective vulnerability. Pioneers like Virginia Lee and John Trojanowski have been instrumental in identifying key proteins (tau, alpha-synuclein, TDP-43) central to these diseases. The synaptic spread hypothesis suggests a cell-to-cell propagation of pathogenic proteins across neuronal synapses, influencing disease progression, with studies highlighting the role of proteins like alpha-synuclein and amyloid-beta in this process. In contrast, the selective vulnerability hypothesis proposes inherent susceptibility of certain neurons to degeneration due to factors like metabolic stress, leading to protein aggregation. Recent advancements in neuroimaging, especially PET/MRI hybrid imaging, offer new insights into these mechanisms. While both hypotheses offer substantial evidence, their relative contributions to neurodegenerative processes remain to be fully elucidated. This uncertainty underscores the necessity for continued research, with a focus on these hypotheses, to develop effective treatments for these devastating diseases.

## 1 Introduction

The development of more effective treatments for neurodegenerative diseases is a significant challenge in modern medicine. Current projections estimate that there are 6.7 million people in the United States living with Alzheimer’s disease (Alzheimer’s Association Report, 2023), as many as 1 million with Parkinson’s disease (U.S. Department of Health and Human Services, 2021), 32,000 with amyotrophic lateral sclerosis (ALS) (Centers for Disease Control and Prevention, 2023).

In the United States, these patients also face high medical costs. The most recent annual report of the Alzheimer’s Association estimates that this disease alone cost Americans $345 billion a year in 2023, over $50,000 a year per patient (Alzheimer’s Association Report, 2023).

The last couple of decades have featured a major conceptual debate within the field of neurodegenerative disease research, one with significant ramifications on the direction of future research efforts and treatment options. The two hypotheses associated with this debate, the synaptic spread hypothesis and the selective vulnerability hypothesis, suggest different potential mechanisms through which degeneration occurs.

## 2 Milestones in neurodegenerative disease research

John Trojanowski and Virginia Lee, both long affiliated with the University of Pennsylvania (Lee still and Trojanowski until his death in 2022), are regarded as major players within the field of neurodegenerative disease research (Huntington’s Disease Society of America, 2020; Brettschneider et al., 2015). John Trojanowski’s first paper on neurodegeneration was published in 1988; he looked at neurofibrillary tangles in the hippocampus of human subjects with and without Alzheimer’s disease (Schmidt et al., 1988). In 1991, Lee and Trojanowski demonstrated that tau, a cytoskeletal protein first isolated by Murray Weingarten and Marc Kirschner in 1975 (Weingarten et al., 1975), was a building block in the neurofibrillary tangles that are often associated with Alzheimer’s disease (Lee et al., 1991).

This finding gave Alzheimer’s researchers a new, specific protein to focus on. The association between mutations to amyloid-beta precursor protein and Alzheimer’s disease was independently hypothesized in that same year (Chartier-Harlin et al., 1991) (the association between plaques and Alzheimer’s dates back over one hundred years (Hippius and Neundörfer, 2003), while the amyloid beta protein fragment was first isolated in 1984) (Glenner and Wong, 1984).

In 1997, working with Maria Spillantini of the University of Cambridge, Lee and Trojanowski co-authored a paper in *Nature* that identified a protein called alpha-synuclein as a major component of a type of protein aggregate called Lewy bodies, these latter structures considered to be characteristic of Parkinson’s pathology (Spillantini et al., 1997).

In the 2007 edition of the *Annals of Neurology*, Lee and Trojanowski published a paper suggesting that a ubiquitinated version of a protein called TDP-43 was a pathological hallmark of sporadic ALS, ALS with dementia, and *SOD1*-negative familial ALS (Mackenzie et al., 2007).

## 3 An overview of the synaptic spread hypothesis and the associated proteins

In 2015, Lee and Trojanowski co-authored a *Nature Reviews Neuroscience* article outlining specific support for the synaptic spread hypothesis (Brettschneider et al., 2015). The paper was co-written by Johannes Brettschneider, who has researched alpha-synuclein pathology in multiple system atrophy (MSA) with Lee and Trojanowski.

The synaptic spread hypothesis suggests cell-to-cell propagation of disease-related proteins in the brains of patients with neurodegenerative disease. The synaptic connections between cells play a large role in determining what areas of the brain sequentially degenerate, although the authors acknowledge that other intrinsic characteristics of the cells themselves, such as their gene expression and their morphology, likely also play a role.

The proteins identified as disease-related are thought to spread across neuronal synapses in the synaptic spread hypothesis. As described in the table, several of the wild type protein and pathological proteins are often found in different locations. These include alpha synuclein, which is found in the presynaptic space as a wild type protein and in the cytoplasm pathologically, amyloid-beta, which is found in the transmembrane space as a wild type protein and in the extracellular space, and TDP-43, which is found in the nucleus as a wild type protein and in the cytoplasm pathologically. While this would potentially fit with a transmission-based pathological process, it is worth noting that tau is found in the cytoplasm both in a wild type and in pathological states such as Alzheimer’s and Pick disease.

**FIGURE 1:**
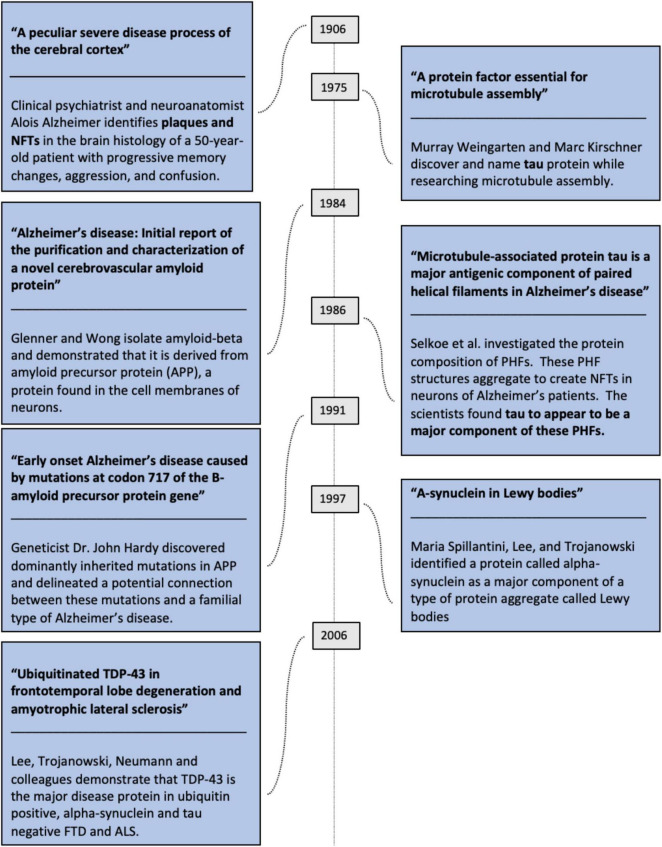
Summation of advances in neurodegenerative disease research.

Thus, the validity of this hypothesis rests on these proteins eventually being properly identified. Here, we will specifically explore (Brettschneider et al., 2015) citations on alpha synuclein, amyloid-beta, tau, and TDP-43, as they are the proteins most emphasized in this paper. All of these theorized protein-disease connections are summarized in Figure 1.

The authors’ citation for the role that the protein alpha-synuclein plays in Parkinson’s disease and Dementia with Lewy Bodies comes from a paper published in a 1998 edition of the *Proceedings of the National Academy of Sciences of the United States of America* authored by Spillantini, Crowther, Jakes, Hasegawa, and Goedert (Spillantini et al., 1998). These researchers had stained for, and found, alpha-synuclein in the neurites of patients with Parkinson’s and in patients with Dementia with Lewy Bodies. Spillantini, Jakes, and Goedert had all co-authored, with Lee and Trojanowski, the landmark 1997 paper that first identified alpha-synuclein in Lewy Bodies (Spillantini et al., 1997).

The authors’ citation for the role that amyloid-beta protein fragments play in Alzheimer’s disease comes from the seminal 1984 paper by pathologist George Glenner and Caine Wong (Glenner and Wong, 1984).

Glenner and Wong isolated amyloid-beta and demonstrated that it is derived from amyloid precursor protein (APP), a protein found in the cell membranes of neurons (Glenner and Wong, 1984). This hypothesis did not gain widespread attention until 1991, when dominantly inherited mutations of APP were discovered by geneticist John Hardy of University College London (Chartier-Harlin et al., 1991). Dr. Hardy delineated a potential connection between these mutations and a familial type of Alzheimer’s disease (Chartier-Harlin et al., 1991).

Research has shown that these amyloid-beta fragments can accumulate into bigger structures called oligomers, leading to the insoluble fibrils and plaques considered to be characteristic of Alzheimer’s disease (Chen et al., 2017).

The authors use three primary citations to argue that the protein tau plays an important role in neurodegenerative diseases. The first of these papers was published by Kosik, Joachim, and Selkoe in a 1986 edition of the *Proceedings of the National Academy of Sciences of the United States of America* (Kosik et al., 1986). These scientists investigated the protein compositions of paired helical filaments (PHFs) found in human neurons. These PHF structures had previously been observed to accumulate in neurons in Alzheimer’s disease and aging processes. Through the utilization of antibody staining, the scientists found tau to appear to be a major component of these PHFs. The authors also cite a 2001 article in the journal *Annual Review of Neuroscience* entitled “Neurodegenerative Tauopathies” written by Lee, Trojanowski, and Goedert as evidence that tau is important to neurodegenerative disease pathologies (Lee et al., 2001). This article details the types of tau inclusions found in Alzheimer’s and frontotemporal lobe dementia (FTLD) patients, as well as the genetic risk factors associated with these diseases. Furthermore, the authors cite a landmark 2013 paper published by Ann McKee and colleagues that found characteristic tau inclusions in the brains of an 85 subject population suspected of having chronic traumatic encephalopathy (CTE) (McKee et al., 2012).

The authors’ citation for the importance of TDP-43 in neurodegenerative diseases is the 2006 *Science* paper that Lee and Trojanowski wrote with Manuela Neumann and colleagues. Here, the researchers found ubiquitinated TDP-43 inclusions in the brains of patients with ALS and FTLD (Neumann et al., 2006).

Table 1 provides us with an overview of where these proteins have mainly been localized in the brain, with and without their associated neurodegenerative disease (Brettschneider et al., 2015). Of the four proteins discussed, alpha-synuclein tends to be found presynaptically in wild-type brains and cytoplasmically in diseased brains, amyloid-beta tends to be found in an transmembrane fashion as part of APP in wild-type brains and is mostly found extracellularly in diseased ones, tau is found cytoplasmically in both wild-type and diseased brains, and TDP-43 is found nuclearly in wild-type brains and cytoplasmically in diseased brains (Brettschneider et al., 2015).

**TABLE 1:** Proteins and their localizations, as thought to be linked to neurodegenerative diseases.

Protein	Human disease associations	Localization of wild type protein	Localization of pathological protein
Alpha-Synuclein	Dementia with Lewy bodies Multiple system atrophy Parkinson’s disease	Presynaptic space	Cytoplasm
Amyloid-Beta	Alzheimer’s disease	Transmembrane	Extracellular space
Tau	Alzheimer’s disease Corticobasal degeneration Pick disease Progressive supranuclear palsy	Cytoplasm	Cytoplasm
TDP-43	Alzheimer’s disease Amyotrophic lateral sclerosis Frontotemporal lobe degeneration	Nucleus	Cytoplasm

Based of summary provided in Brettschneider et al. (2015).

## 4 A dive into the synaptic spread hypothesis

As is explained in the review by Brettschneider et al., some of the earliest evidence that neurodegenerative diseases may involve the spread of pathogenic proteins came from the 2008 work of Professor Jeffrey Kordower, of the Rush University Medical Center in Chicago, and colleagues. Kordower published a pair of case reports (Kordower et al., 2008a; Kordower et al., 2008b) in 2008 investigating (post-mortem) the brains of patients who had participated in an open clinical trial run by Dr. Curt Freed of the University of Colorado School of Medicine. Freed had published his initial findings in a 2001 edition of *The New England Journal of Medicine* (Freed et al., 2001).

In the clinical trial, Freed and colleagues had taken a population of 40 patients, ranging in age from 34 to 75, with severe Parkinson’s disease and assigned them randomly to two different groups. One of these groups was to receive a transplant of cultured brain tissue taken from the mesencephalon (midbrain) of four different embryos. This tissue was to be placed bilaterally into the putamen during open brain surgery. The other group of patients underwent a sham surgery in which holes were drilled into their skulls but no tissue was transplanted. The metrics for this study were largely determined by the presence or absence of statistically significant differences between the groups on subjective rating scales such as the Unified Parkinson’s Disease Rating Scale (UPDRS) and the Schwab and England scale. The researchers also used positron-emission tomography to examine differences in neuron fiber outgrowth between the two groups.

While the researchers found some improvement in symptoms in patients under the age of 60 in UPDRS (*p* = 0.01) and Schwab and England (*p* = 0.06), there was no statistical improvement in older patients. Further, after improvement in the first year, 15% of patients experienced recurrence of dystonia and dyskinesia. Further, as was strongly emphasized in a *New York Times* article written by Gina Kolata on the clinical trial, a number of patients, including younger ones, had salient, negative side effects in response to the transplantation (Kolata, 2001). These side effects largely consisted of jerky, uncontrollable movements in about 15% of patients, with the researchers advising 6 of the patients in the study who had not yet received an implant to go without it. Dr. Greene, an author on the study, described the side effects as “absolutely devastating,” noting that they could not be turned off, and summarized his positions as “no more fetal transplant” (Kolata, 2001).

Even though the clinical trial seemed to have limited clinical application at the time, Kordower’s investigation of the brains of two patients who had received transplantations in the Freed trial proved to be hugely important in helping shape the synaptic spread hypothesis. Indeed, in both cases, the transplanted neurons, despite their youth, seemed to exhibit Parkinson’s pathology. In the article “Transplanted Dopaminergic Neurons Develop PD Pathologic Changes: A Second Case Report,” published by Kordower and colleagues in *Movement Disorders*, Parkinson’s-type pathology is described in the grafted neurons of patient MK (Kordower et al., 2008b). Patient MK was 38 when he was diagnosed with Parkinson’s disease and he had the diagnosis for 25 years before he received the transplantation. Despite a good initial response to levodopa, Patient MK was afflicted with the severe motor side effects and died 14 years after the procedure.

In an autopsy conducted 3 h after MK’s death, researchers found that the grafted neurons had formed a large number of axonal connections to the putamen (Kordower et al., 2008b). This is important to understand in relation to the article’s support for the synaptic spread hypothesis, which dictates that disease proteins spread across synapses. Stainings of the grafted neurons found a number that stained positive for alpha-synuclein, ubiquitin, and thioflavin-S, described as being nearly identical in morphology and staining to the Lewy bodies associated with Parkinson’s pathology. Additional stainings of these grafted cells found that many of them no longer expressed the dopamine transporter (DAT).

These findings may seem surprising, as these grafted neurons were only fourteen years in age, an age group in which Parkinson’s is exceedingly rare in humans. As opposed to the selective vulnerability hypothesis, which states that the most disease-vulnerable neurons degenerate first followed sequentially by moderately disease-vulnerable neurons and then by less disease-vulnerable neurons, the synaptic spread hypothesis seems to make more sense here. Given the youth of the transplanted neurons, the fact that so many synapses formed between the grafted neurons and the diseased putamen seems to indicate that there is a possibility that there was some pathogenic protein spread across synapses. However, this process was not directly deserved and it remains possible that there is some other factor of the originally diseased neurons that afflicted the grafted neurons without any sort of synaptic transmission.

Kordower reported very similar results in his other case report, published in a 2008 edition of *Nature Medicine* (Kordower et al., 2008a). This case report investigated a female patient who had received the transplantation at the age of 61, 22 years after her diagnosis of Parkinson’s disease. Like patient MK, she had lived 14 years after the transplantation, dying in 2007. This patient improved by most metrics after the surgery and did not report any serious side effects until her disease rapidly progressed in 2004 before her death by cardiac arrest three years later.

Stainings done in the brain of this patient exhibited reduced DAT expression. Many of this patient’s grafted cells also stained positive for “aggregated and neuritic alpha-synuclein,” (Kordower et al., 2008a) findings indicative of Parkinson’s-type pathology. Just as in patient MK, no direct synaptic transmission of disease proteins was found in this patient, and such a process would not likely be found in an autopsy. However, the fact that disease-type pathology was found in the grafted neurons despite their youth hints at the possibility of the synaptic spread hypothesis over the selective vulnerability one.

In order to advance the synaptic spread hypothesis, Brettschneider et al. provide an overview of how pathogenic proteins might spread across synapses (Brettschneider et al., 2015). They begin this discussion by trying to answer a major question: how do proteins become pathogenic in the first place? They thus introduce us to the idea of template-directed misfolding, in which a protein that has been misfolded interacts with a healthy, “native” protein and causes this latter protein to also become misfolded. Their evidence for this includes a 2011 *in vitro* study by Grad of human neural and mesenchymal (a type of multipotent stem cell) cell lines in which a mutant version of the SOD1 protein was observed to induce the misfolding of native SOD1 protein (Grad et al., 2011).

After template-directed misfolding, the authors suggest that the next step in forming neurodegenerative disease is seeded aggregation (Brettschneider et al., 2015). In this process, with more and more template-directed misfolding events happening and more and more misfolded proteins accumulating, aggregates of proteins begin to form. Early evidence for this came from Baker and colleagues in 1993, when seeded aggregation of amyloid beta was observed in marmosets (Baker et al., 1993).

Seeded aggregation has been a topic of much research in the decades since. A 2009 study by Florence Clavaguera of the University of Basel found that when transgenic mice that initially expressed wild type human tau received an injection of mutant human tau, a progressive seeding aggregation process followed (Clavaguera et al., 2009). This seeding aggregation process has even been seen using synthetic, mutant-like tau injections, having been observed by Guo and Lee in 2013 in human cells (Guo et al., 2013).

**FIGURE 2:**
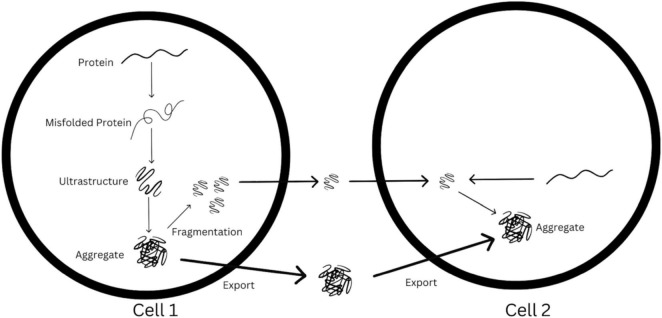
Visualization of possible synaptic spread mechanism from cell 1 to cell 2, based loosely off of original figure in Brettschneider et al. (2015).

The steps of template-directed misfolding and seeded aggregation are represented in diagram-fashion in Figure 2. As Figure 2 demonstrates, the synaptic spread hypothesis argues that when enough of these seeds are aggregated, they are released into the synapse, they travel towards another cell, and are uptaken by this other cell. In this new cell, there is further seeded aggregation, leading to the accumulation of misfolded protein in this cell. If this process happens many times over, synaptic transmission could lead to these pathogenic protein deposits being present across vast areas of the brain.

Brettschneider et al. do indeed try to provide evidence of a mechanism of spreading. For one, they cite a 2011 study that seems to show that fibrillar alpha-synuclein can spread both anterogradely through axons to secord-order in *in vitro* cell cultures (Freundt et al., 2012). Studies in cultured neurons on TDP-43, specifically the 2012 paper by Fallini et al. published in *Human Molecular Genetics* (Fallini et al., 2012), and on tau, such as the 2002 *Journal of Neuroscience* paper published by Utton et al. (2002), indicate anterograde movement of these proteins through axons.

Further support for the synaptic spread hypothesis comes from a pair of mouse studies. In one, published by Iba and colleagues in a 2013 edition of the *Journal of Neuroscience*, injections of tau fibrils into transgenic mice who expressed mutant human tau led to protein aggregates in areas of the brain that were far removed from the injection site (Iba et al., 2013). Brettschneider et al. argue that this indicates that “neuronal connections are probably involved in disease protein propagation and disease spread.” A 2012 study by Luk et al. published in the *Journal of Experimental Medicine*, in which injections of alpha-synuclein fibrils were given to transgenic mice who expressed mutant human alpha-synuclein also showed protein accumulations in areas far from the injection site (Luk et al., 2012), with the synaptic spread hypothesis being a possibility for this dissemination.

The literature review by Brettschneider et al. does provide us with some important evidence that cellular uptake of tau and alpha-synuclein have been observed via fluid-phase and receptor-mediated endocytosis. This evidence includes a 2013 paper published in the *Journal of Biological Chemistry* by Wu et al. (2013) that demonstrated that small, misfolded tau can be uptaken by cells from a mouse neuronal cell line through a process of endocytosis and can then be translated retrogradely and anterogradely in neurons. Wu was able to characterize this process as bulk endocytosis, in which a particularly large portion of the presynaptic plasma membrane buds off to form the endosome.

A 2008 paper published by Lee and colleagues in the *International Journal of Biochemistry and Cell Biology* (Lee et al., 2008) found that cells in a cortical neuronal cell line taken originally from a rat embryo were able to uptake alpha-synuclein that was initially outside of the cells. While these studies show that animal cells seem to be capable of taking up potentially pathogenic proteins from extracellular space, we must keep in mind that both of these studies were *in vitro* and with non-human cell lines.

A careful examination of Figure 2 and Brettschneider et al. (2015) reveals something important about the synaptic spread hypothesis as defined by Brettschneider et al.; these authors claim that disease proteins can spread across synapses both to neighboring neurons and to glial cells. Here, it is worth reminding ourselves that neuron-glia synapses do occur in the brain (Bergles et al., 2010), and so the synaptic transmission of these proteins could very well carry them into glial cells.

Evidence for this transmission, however, seems to be even more tentative than that for neuron-neuron transmission. A 2020 review by Kaji et al. (2020) indicates that current evidence suggests that glial inclusions precedes general neurodegeneration in multiple system atrophy. Glia remain an exciting field of research in neurodegeneration, with a 2021 editorial by Bennett and Sloan recognizing that while much research has focused on microglia and astrocytes, there is exciting research being done now in pericytes and even meningeal immune cells (Bennett and Sloan, 2021).

## 5 Critiques and a dive into the selective vulnerability hypothesis

A critique of the synaptic spread hypothesis that also touches in greater depth on the selective vulnerability hypothesis was authored in a 2016 edition of *Nature Reviews Neuroscience* by Dominic Walsh and Dennis Selkoe (Walsh and Selkoe, 2016).

Walsh and Selkoe certainly do not rule out the synaptic spread hypothesis in their paper, instead just cautioning us to remind ourselves that this process has never been directly observed in a human being with a neurodegenerative disease. Walsh and Selkoe also push against the concept promoted by Brettschneider et al. that neurodegenerative diseases can mechanistically be explained by a prion-like, self-propagating mechanism focused on progressive seeded aggregation (Brettschneider et al., 2015). This description, Walsh and Selkoe argue, is far too precise given current knowledge.

Walsh and Selkoe flesh out the selective vulnerability hypothesis much more fully. They explain to us that in response to certain unfavorable circumstances, giving the example of external stress, proteins start to aggregate in neurons that are especially vulnerable to these circumstances. Over time, these protein aggregates are seen in other cells that are perhaps only moderately vulnerable to these adverse circumstances, followed by cells that are perhaps not as vulnerable to these circumstances.

Their description also reminds us that even if it is not proteins that are spreading synaptically from cell-to-cell, there are other things that could be transferred from cell to cell that could cause disease. As an example, they discuss how metabolic factors could diffuse across the synapse and be taken up by a neighboring neuron, which then could have a metabolic issue that resulted in protein aggregates. Walsh and Selkoe emphasize to us that the synaptic spread and selective vulnerability hypotheses are not mutually exclusive; both diffusible metabolic factors and pathogenic proteins could be transferred synaptically and both could be implicated in causing disease-related physiological changes.

One citation in Walsh and Selkoe that explicitly references the selective vulnerability hypothesis is a paper by Walker Jackson, of Linköping University in Sweden, published in 2014 in *Disease Models and Mechanisms* (Jackson, 2014). One potential reason that is suggested by Jackson as to why some regions of the brain appear to be more vulnerable to disease pathology is that perhaps these areas of the brain just express more levels of the protein in the first place. Jackson quickly dismisses this explanation, however, as several studies on genes related to neurodegenerative disease have found that the genes are similarly expressed in areas of the brain both affected and seemingly spared by the illness.

Jackson thinks that a more plausible explanation is that the cells in the brain with intrinsically higher firing rates are more likely to degenerate. One explanation he posits for this is that more active cells could be damaged more frequently by free radicals. He cites a 2007 study published by Chan and colleagues in *Nature* that found that in a transgenic mouse model, Parkinson’s-type pathology found that the substantia nigra cells rely on calcium channels with high firing rates (Chan et al., 2007).

Jackson theorizes that metal ions may serve an important role in determining the vulnerability of certain brain regions to neurodegenerative disease, reminding us that an appropriate stoichiometric balance of metals and biomolecules is critical for a number of syntheses and degradation processes. As discussed in a 2023 review by Yoo et al. (2023), transition metals can bind to a variety of neurotransmitters, including dopamine, serotonin, and epinephrine. Much research has associated the involvement of these metals with different neurodegenerative disease processes including Alzheimer’s (Nam et al., 2018) and Parkinson’s (Mahoney-Sánchez et al., 2021).

Walsh and Selkoe are critical of the notion espoused by Brettschneider et al. that the Braak staging system of Alzheimer’s disease provides evidence for the synaptic transmission of pathogenic proteins (Walsh and Selkoe, 2016). It is unfair, Walsh and Selkoe argue, to look at the brains of many people with Alzheimer’s disease who have died at many different ages and to stage these people’s diseases post-mortem based on protein spread. This is because, as is detailed in a 2004 paper by Avila and colleagues in *Physiology Reviews* (Avila et al., 2004), these proteins are implicated in several different diseases. Given the diversity of this patient group, it would have to be very thoroughly determined that these protein deposits were from Alzheimer’s disease and not something else. Further, even if all of these protein deposits were from Alzheimer’s disease, it would still have to be conclusively determined that this was the result of synaptic-driven protein spread and not the spread of metabolic factors or another material.

Walsh and Selkoe also alert us to a finding published by Heiko Braak and Kelly Del Tredici in 2011 in *Acta Neuropathologica* (Braak and Del Tredici, 2010). These researchers found small neurofibrillary changes in the locus coeruleus of a child who had died at 6 and in another who had died at 14. While Braak and Del Tredici took these findings to indicate that these children would likely have gone on to develop Alzheimer’s disease later in life, Walsh and Selkoe are skeptical. They remind us that findings such as aggregates of the tau protein cannot be linked specifically to a single disease, because these aggregates are, in fact, associated with several different diseases.

In their critique of the synaptic spread hypothesis, (Walsh and Selkoe, 2016) also are somewhat critical of the notion that Kordower’s autopsies of patients (Kordower et al., 2008a; Kordower et al., 2008b) from the Freed trial (Freed et al., 2001) provide strong support for that hypothesis. They emphasize that the fact that Lewy bodies were detected in about 5–10% of the grafted cells would likely have few functional ramifications on the subjects. Further, they suggest alternatives for why some grafted neurons may have Lewy bodies, including the possibility that a process such as astrocytosis or microgliosis could have caused the uptake of alpha-synuclein in a grafted cell.

The review by Selkoe and Walsh is also critical of some of the animal model studies cited by Brettschneider et al., including the previously described 2012 Luk article (Luk et al., 2012) that demonstrated that injections of high levels of recombinant alpha-synuclein into mice can lead to the formation of Lewy bodies in these mice. They point out that these experiments used artificially inflated levels of pre-formed fibrils of alpha-synuclein, meaning that any connections to disease in humans, who have lower levels of the alpha-synuclein protein, should be considered tentative. Another animal study cited by Brettschneider et al., published in 2014 in the *Annals of Neurology* by Recasens et al. (2014) found that rhesus monkeys who had received injections of Parkinson’s-derived Lewy bodies show evidence of disease histology as well as reduced nigrostriatal innervation as determined by PET scan. Selkoe and Walsh warn us, once again, that these results are preliminary, as they were taken from the brains of just four animals. However, they do seem excited by this study, as it provides evidence of seeded aggregation in monkeys, animals much more closely related to humans than mice and rats are. The scientists thus call for more primate research into Parkinson’s and Alzheimer’s disease.

Legend: 1. Traveling exosomes, 2. Tunneling nanotubules between cells, 3. Raw protein release from synapse, 4. Stress release of proteins.

**FIGURE 3:**
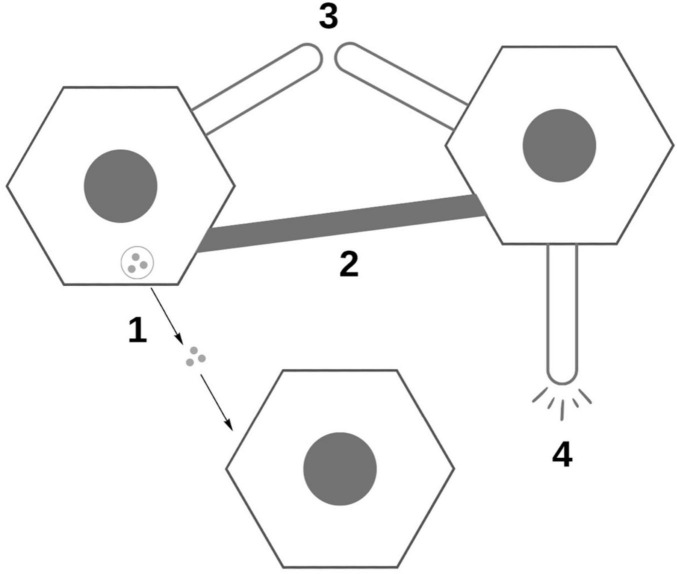
Visualization of diversity of potential mechanisms for synaptic spread, based off of diagram in Walsh and Selkoe (2016).

A final convincing point for Selkoe and Walsh’s argument that we do not yet know enough to convincingly declare the synaptic spread hypothesis to be true lies in the figure that is included as Figure 3 in this paper (Walsh and Selkoe, 2016). Figure 3 illustrates four different mechanisms (labeled 1–4 in the diagram) as to how exactly proteins could be transferred from cell to cell. Each is strikingly different. This all shows us just how much we do not know when it comes to the synaptic spread hypothesis. The first mechanism, as it is labeled, suggests that these proteins travel between cells in little vesicles referred to as exosomes. A second mechanism theorizes that the proteins travel across thin bridges called tunneling nanotubes (TnTs) that form between cells. A third mechanism posits that small amounts of the raw, naked protein could “leak” between the presynaptic and postsynaptic endings, illustrated here as being across a short distance. The final mechanism in this diagram suggests that these proteins are released by diseased cells in response to biological stress and are then taken up by another cell.

Figure 3 seems to capture the essence of Walsh and Selkoe’s paper. Walsh and Selkoe are not, after all, innately opposed to the idea of the synaptic spread hypothesis. Instead, their opinion is that this hypothesis and the selective vulnerability one are likely to both be at play, with the individual extent of each still yet to be fully discovered. This is in line with Walsh and Selkoe’s calling for greater research into both the synaptic spread and selective vulnerability hypotheses.

## 6 PET/MRI imaging and neurodegenerative disease

Recent advances in the field of imaging provide promise for further investigation into the synaptic spread and selective vulnerability hypotheses. One such advance has been the development of new radiotracers for the investigation of neural activity. While (Lee et al., 2001)-2-fluoro-D-glucose (FDG) has been used since 1979 (Phelps et al., 1979), there are now also tracers for amyloid beta (Krishnadas et al., 2021) and tau (Okamura et al., 2005).

Another such advance is that of the PET/MRI hybrid imaging, a technique that was first conceived of in the 1980s (Mannheim et al., 2018). As this review by Mannheim et al. describes (Mannheim et al., 2018), while PET imaging has sensitivity into the range of picomolar concentrations and is excellent for the investigation of biomolecular probes, MRI has excellent structural and anatomical resolution. The process of coregistering MRI and PET images to a standard template was a process that first began in the 1990s (Meltzer et al., 1996), allowing the analysis of data from both MRI and PET. However, this process of coregistration was not without its downsides, requiring significant processing and lacked an appropriate set of tools to localize and estimate error and distortion (Crum et al., 2003).

The simultaneous imaging of a subject with an MRI and PET scan can overcome some of these shortcomings with the MRI and PET data being collected both at the same time and in the same space (Tondo et al., 2019). This enhanced level of the detail, with the potential for biomolecular and structural precision, could be the key to providing granular, in vivo evidence of the synaptic spread and selective vulnerability mechanisms. However, as described in the review by Tondo et al., this combined technology is still new enough that these scanners are not nearly as available in both the research and clinical arenas as traditional MRI and PET scanners.

Nevertheless, PET/MRI imaging is already being used in the study of neurodegenerative disease, with its spatial and biomolecular precision already offering promise. A study by Marchitelli et al. in 2018 used FDG-PET/fMRI scans in the investigation of the brains of 23 individuals with cognitive impairment and 23 health controls (Anazodo et al., 2018). In this study, these scans were able to effectively differentiate these two populations while also showing that abnormal glucose usage is linked to the degeneration found in the dementia process. Hybrid PET/MRI has also been described as being ideal for the investigation of dopamine release in Parkinson’s patients, with the ability to measure dopamine levels at both the presynaptic and postsynaptic areas possible with radiotracers (Tondo et al., 2019).

While still relatively new to neurodegenerative research, the upside of PET/MRI imaging in the evaluation of the synaptic spread and selective vulnerability hypotheses seems quite large.

## 7 Conclusion

An assessment of the synaptic spread and selective vulnerability hypotheses reveals that the field of neuroscience research is not yet at a point where we can conclusively choose one theory over the other as the definitive mechanism of neurodegeneration. In fact, it is likely that both hypotheses are at play in all of these diseases. This, coupled with advances in techniques like neuroimaging, has led to a very exciting time in neurodegenerative disease research in which one can make one’s own assessment of the literature on synaptic transmission and selective vulnerability and then one can carefully choose where to spend one’s time and effort in pursuing a meaningful project. While many researchers want at some level to discover the next great advance, we have learned from the work of Dr. Freed that publishing seemingly negative results is quite important (Freed et al., 2001). Therefore, we strongly advocate that scientists vigorously continue their research efforts into both the synaptic spread and selective vulnerability hypotheses. Ultimately, the authors of this review view further research into the synaptic spread and selective vulnerability hypotheses as both being critically important in bringing relief to those millions of people who suffer from neurodegenerative diseases. While conducting and critically evaluating this research is certainly time and energy intensive, this work is ultimately all for them.
